# Quorum Sensing in the Squid-*Vibrio* Symbiosis

**DOI:** 10.3390/ijms140816386

**Published:** 2013-08-07

**Authors:** Subhash C. Verma, Tim Miyashiro

**Affiliations:** Department of Biochemistry and Molecular Biology, Eberly College of Science, the Pennsylvania State University, 219 Wartik Lab, University Park, PA 16802, USA; E-Mail: scv1@psu.edu

**Keywords:** quorum sensing, symbiosis, *Vibrio fischeri*, *Euprymna scolopes*, luminescence, motility

## Abstract

Quorum sensing is an intercellular form of communication that bacteria use to coordinate group behaviors such as biofilm formation and the production of antibiotics and virulence factors. The term quorum sensing was originally coined to describe the mechanism underlying the onset of luminescence production in cultures of the marine bacterium *Vibrio fischeri*. Luminescence and, more generally, quorum sensing are important for *V. fischeri* to form a mutualistic symbiosis with the Hawaiian bobtail squid, *Euprymna scolopes.* The symbiosis is established when *V. fischeri* cells migrate via flagella-based motility from the surrounding seawater into a specialized structure injuvenile squid called the light organ. The cells grow to high cell densities within the light organ where the infection persists over the lifetime of the animal. A hallmark of a successful symbiosis is the luminescence produced by *V. fischeri* that camouflages the squid at night by eliminating its shadow within the water column. While the regulatory networks governing quorum sensing are critical for properly regulating *V. fischeri* luminescence within the squid light organ, they also regulate luminescence-independent processes during symbiosis. In this review, we discuss the quorum-sensing network of *V. fischeri* and highlight its impact at various stages during host colonization.

## 1. Introduction

### 1.1. Quorum Sensing in *Vibrio fischeri*

Quorum sensing (QS) describes the mechanism of intercellular communication by which bacteria can alter group behavior in accordance with population density [1]. This process depends on the synthesis and diffusion of signaling molecules, called autoinducers, into the surrounding environment. Upon reaching a threshold concentration, autoinducers will trigger cellular responses, typically by altering gene expression across the entire population. QS controls a wide variety of processes in bacteria including bioluminescence production, sporulation, competence, biofilm formation and the synthesis of antibiotics and virulence factors [2,3].

QS was first discovered in the Gram-negative, marine bacterium *Vibrio fischeri* as the mechanism that controls the induction of luminescence within growing cultures [4]. *V. fischeri* belongs to the family *Vibrionaceae*, which is comprised of many bacterial species that are commonly found in fresh and marine water habitats. *Vibrio fischeri* was originally described as a member of the genus *Vibrio*. More recently, however, *Vibrio fischeri*, along with *Vibrio logei*, *Vibrio salmonicida*, and *Vibrio wodanis*, has been placed in a new genus called *Aliivibrio*, as these species form a monophyletic clade that can be differentiated based on phenotypic traits and biochemical tests from the other members of the genus [5]. Because the majority of studies focusing on QS and host-microbe interactions use the *Vibrio* nomenclature, we will continue its use in this review.

The proteins required for luminescence in *V. fischeri* are encoded within the *luxICDABEG* operon [6,7]. Light is released during the oxidation reaction by the enzyme luciferase, which is comprised of two subunits, α and β, encoded by *luxA* and *luxB*, respectively. The enzymatic reaction converts a long-chain fatty acid (RCHO), reduced flavin mononucleotide (FMNH_2_), and O_2_ into aliphatic acid (RCOOH) and FMN. A reductase complex composed of the proteins encoded by *luxCDE* synthesizes the substrate RCHO [8]. LuxG converts FMN to FMNH_2_ [9].

**Figure 1:**
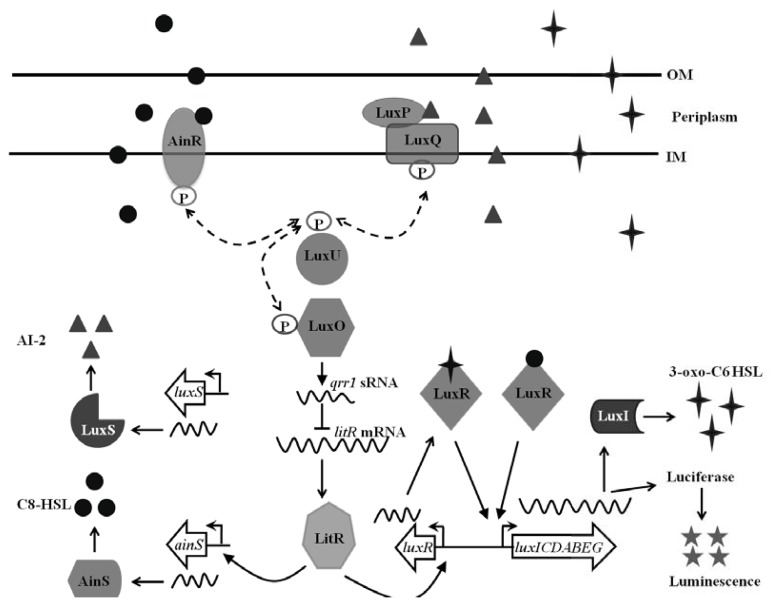
The QS network of *V. fischeri. V. fischeri* has three QS systems: LuxI-LuxR, AinS-AinR, and LuxS-LuxP/Q. In the absence of C8-HSL and AI-2 autoinducers, LuxO is phosphorylated by the kinase activities of the histidine kinases AinR and LuxQ. Phosphorylated LuxO activates expression of the sRNA Qrr1, which degrades via Hfq the mRNA of *litR*, thereby reducing the level of the transcription factor LitR. Accumulation of C8-HSL and AI-2 at high cell density results in decreased phosphorylation of LuxO, which enhances the level of LitR. LitR activates transcription of *luxR*, which encodes the transcription factor that, when bound by the autoinducer 3-oxo-C6-HSL, directly regulates expression of the luminescence (*lux*) genes. C8-HSL can also affect luminescence by directly binding to LuxR. The LuxR/C8-HSL complex can activate transcription of the *lux* genes, although less effectively than the LuxR/3-oxo-C6-HSL complex. In addition to encoding the light-producing enzyme luciferase, the *lux* operon contains *luxI*, which encodes the synthase LuxI that synthesizes 3-oxo-C6-HSL. As described in the main text, synthesis of both C8-HSL and 3-oxo-C6-HSL is autoregulated by separate positive feedback loops. OM = outer membrane, IM = inner membrane.

Multiple QS systems control luminescence in *V. fischeri* (Figure 1). Directly regulating expression of the *luxICDABEG* operon is the LuxI-LuxR QS system. LuxI synthesizes the autoinducer *N*-3-oxohexanoyl-homoserine lactone (3-oxo-C6-HSL) [10] that, at a threshold concentration (100–200 nM), binds and activates the transcription factor LuxR [11]. The LuxR/3-oxo-C6-HSL complex binds as a dimer upstream of the *luxICDABEG* operon and recruits RNA polymerase to initiate transcription of the operon [12]. Interestingly, while the overall luminescence output of a population of cells increases as the level of 3-oxo-C6-HSL increases, the luminescence levels of individual cells display significant heterogeneity [13]. Whether this cell-cell heterogeneity in luminescence output has a biological role remains unclear at this time.

Two additional QS systems, AinS-AinR and LuxS-LuxP/Q, indirectly control luminescence by modulating *luxR* transcription (Figure 1). AinS synthesizes *N*-octanoyl-homoserine lactone (C8-HSL), which is an autoinducer detected by the histidine kinase AinR. LuxS synthesizes autoinducer 2 (AI-2), which binds to the periplasmic protein LuxP [14,15]. Based on studies of the homologous system in *V. harveyi*, LuxP forms a complex with LuxQ, a histidine kinase that dimerizes within the inner membrane [15]. Binding of AI-2 to LuxP induces a rotational shift in the corresponding dimer of LuxQ that inhibits kinase activity. AinR and LuxP/Q act in parallel by controlling the phosphorylation state of LuxU, which serves as an intermediate step within the phosphorelay that dictates the phosphorylation state, and consequently, activity of the response regulator LuxO. Under conditions of low cell density, *i.e.*, in the absence of sufficient levels of autoinducers, the histidine kinases activate the phosphorelay, leading to high levels of phosphorylated LuxO. Phosphorylated LuxO activates transcription of *qrr1*, which encodes a small regulatory RNA that post-transcriptionally represses the transcription factor LitR via the RNA chaperone Hfq [16]. Under conditions of high cell density, the phosphorelay is reversed, which stabilizes *litR* transcript. LitR enhances *luxR* expression, thereby contributing to light production [17].

The QS network of *V. fischeri* is significantly different than those of other *Vibrionaceae* members [18]. For instance, the LuxI-LuxR system is exclusively present in the *Aliivibrio* clade of the *Vibrionaceae* family, which includes the fish pathogen *Aliivibrio salmonicida* as well as *Vibrio fischeri* [19]. In non-*Aliivibrio* members of *Vibrionaceae* that are bioluminescent, the luminescence genes are directly regulated by the corresponding LitR homologue instead of a LuxI-LuxR system. As a result, a distinguishing feature of the *V. fischeri* QS network is that, in contrast to parallel QS networks in *V. harveyi* and *V. cholerae*, it is both parallel and hierarchal. In other words, in all *Vibrionaceae* members, histidine kinases, like AinS-AinR and LuxS-LuxP/Q in *V. fischeri*, are arranged in parallel within the network and converge on the regulator LitR. The *V. fischeri* network is also hierarchal because the LuxI-R system acts downstream of LitR, and therefore depends, in part, on the activities of the parallel QS systems. Another significant difference among the QS systems of different *Vibrionaceae* members is the number of *qrr* genes within their genomes. In particular, *V. fischeri* possesses a single *qrr* gene encoding Qrr1 that is sufficient to repress *litR* expression [16], whereas multiple Qrr sRNAs regulate the expression of the LitR homologues in *V. harveyi* (LuxR_VH_) and *V. cholerae* (HapR) [20,21].

In *V. fischeri*, the QS network also appears to have crosstalk among the different autoinducers: namely, C8-HSL, the autoinducer synthesized by AinS, can also directly bind to LuxR [14,22]. However, the LuxR/C8-HSL complex is not as effective as the LuxR/3-oxo-C6-HSL complex in activating transcription of the *lux* operon [22,23]. As a result, the two autoinducers show competitive binding to LuxR that alters the transcriptional level of the *lux* operon and resulting luminescence. At high concentration, C8-HSL acts as a competitive inhibitor and suppresses luminescence; however, the suppression can be overcome by sufficiently high concentrations of 3-oxo-C6-HSL [14,22]. Interestingly, the impact of deleting *ainS* on luminescence depends on the particular strain of *V. fischeri*, highlighting the complexity by which C8-HSL affects luminescence [14,22]. The crosstalk by autoinducers does not appear to affect the heterogeneity in the luminescence output observed among individual cells [24].

The QS network of *V. fischeri* also contains feedback loops that ultimately impact its overall dynamics. For instance, the LuxI-derived autoinducer 3-oxo-C6-HSL enhances its own synthesis as the *luxI* gene is upregulated by the LuxR/3-oxo-C6-HSL. Interestingly, a positive feedback loop is also involved in QS by C8-HSL, as *ainS* expression is apparently controlled through LitR [25]. How different architectures of various QS networks can impact the magnitudes and temporal profiles of QS-regulated phenotypes is well documented [2,3]. As described throughout this review, the particular intricacies of the QS network of *V. fischeri*, which experiences both free-living and symbiotic conditions, has evolved to accommodate such disparate life styles.

Notably, the QS network of *V. fischeri* regulates processes in addition to bioluminescence. Flagella-based motility is controlled, in part, by the LuxO-Qrr1-LitR pathway, so that motility is enhanced under the conditions of low cell density that repress bioluminescence. Phosphorylation of LuxO, or equivalently expression of *qrr1*, results in enhanced motility. Epistasis experiments have demonstrated that LitR is the transcription factor that mediates the effect on motility by LuxO and Qrr1 [26]. AinS also regulates via LitR the expression of *acs*, which encodes acetyl-CoA synthetase and, as a result, can control acetate metabolism [27]. More recently, the LuxP/Q complex was shown to impact biofilm formation by *V. fischeri*, which is a process mediated by an 18-gene, symbiosis polysaccharide (*syp*) locus [28]. Interestingly, the mechanism may involve direct phosphotransfer between LuxU and the response regulator SypG, although this has yet to be shown.

### 1.2. The *Euprymna scolopes-Vibrio fischeri* Symbiosis

**Figure 2:**
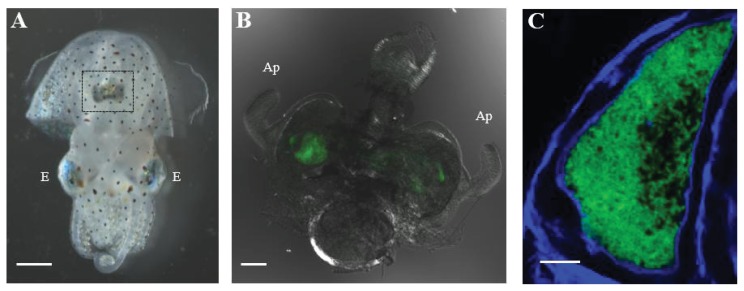
The light organ of a juvenile *E. scolopes* harboring *V. fischeri*. (**A**) Bright field image showing the ventral side of a juvenile *E. scolopes*. The dark structure highlighted in the box is the light organ. Scale bar = 1 mm. E = eye; (**B**) Differential interference contrast (DIC) image of a 48-h p.i. light organ colonized with GFP-labeled *V. fischeri* cells (green). Scale bar = 100 μm. Ap = appendages; (**C**) Confocal image of a light organ crypt colonized with GFP-labeled *V. fischeri* cells (green). Host actin is stained with phalloidin (blue). Scale bar = 10 μm.

Recent studies of the human gut microbiome have highlighted the general importance of beneficial bacteria on animal physiology and development [29]. *V. fischeri* forms monospecific, beneficial symbioses in many marine animals, including various squid and fish [30,31]. Of these hosts, *E. scolopes*, a species of bobtail squid found in the offshore waters of the Hawaiian Islands, is the most studied. The symbiosis is highly specific, such that the squid is colonized exclusively by only certain strains of *V. fischeri* [31,32]. In addition, bacterial transmission is horizontal, *i.e.*, *V. fischeri* cells are acquired each generation by juveniles from seawater. Because of these features, the symbiosis offers an excellent model to study the establishment, development, and maintenance of a beneficial bacterial infection along an epithelial surface [33]. The nocturnal squid uses bioluminescence emitted by *V. fischeri* for camouflage by shining light downwards to disrupt its shadow within the water column [34]. To harness this bioluminescence, *E. scolopes* houses populations of *V. fischeri* within an organ referred to as the light organ that is located in the middle of the mantle cavity, just inside of the ventral surface of the mantle (Figure 2). The light organ possesses bilateral symmetry and, in juvenile squid, exhibits on each side two appendages that are surrounded by a field of ciliated epithelia [35]. At the base of the appendages on either side of the light organ are three pores, which lead through ciliated ducts to crypt spaces deep within the organ, where bacteria reside extracellularly during the lifetime of the host.

The initial process of colonization begins within 2–4 h after juvenile squid hatch from their eggs into seawater containing as little as 1000 *V. fischeri* CFU/mL [36]. The first event in establishing the symbiosis is the direct interaction of bacterial cells, including *V. fischeri*, to host cilia that are associated with surface epithelial cells [37,38]. Beating of these cilia collect bacteria in aggregates, which are comprised of up to a few hundred cells within host-derived mucus that is secreted by surface epithelial cells of the light organ [37]. Interestingly, hyper-motile mutants of *V. fischeri* show a delay in aggregate formation, which highlights the importance of proper regulatory control over motility throughout the colonization process [39]. Although mucus secretion is a general host response to the presence of bacteria [32], the aggregate is dominated by *V. fischeri* cells [40]. The mechanism by which *V. fischeri* dominates the aggregate despite its low abundance in seawater is largely unknown. It is unlikely that *V. fischeri* cells within the aggregate break down the mucus for nutrients more efficiently than others as the cells do not multiply in the aggregate. Chemotaxis towards *N*-acetylneuraminic acid, a component of squid mucus, suggests that *V. fischeri* has evolved to respond to host-derived compounds [41]. After 2–4 h within the aggregate, *V. fischeri* migrates through the mucus towards the pores. Non-motile *V. fischeri* and bacterial-sized particles do not display this behavior, suggesting that *V. fischeri* actively participates in the process [37]. Chemotaxis to host-derived compounds does not play a role during these steps, since a *cheA* transposon-insertion mutant of *V. fischeri* migrates to the pores in similar fashion to wild-type cells [42].

Once *V. fischeri* has entered a pore, it travels through ciliated ducts to gain access to deep crypt spaces, where it begins to multiply and establishes a stable association [33]. Within each duct, a gradient of chitin-derived oligosaccharides, which are chemoattractants for *V. fischeri*, recruit potential symbionts into the crypt spaces [42]. As described above, the effect of this chemotactic gradient seems to be important only for entry into the pores and travelling through the ducts, not for the initial migration through the mucus [42]. Within the deep crypts, *V. fischeri* grows rapidly for the first 12 h, until the population size reaches about 10^5^ CFU [43]. This growth is concomitant with luminescence, which can be detected as early as 7 h and achieves steady-state levels by 12 h [43].

## 2. Impact of QS Network on the *Euprymna scolopes*-*Vibrio fischeri* Symbiosis

### 2.1. Impact of LuxI-LuxR Signaling

Luminescence is critical for the *Euprymna scolopes*-*Vibrio fischeri* symbiosis to persist. The requirement of bacterial luminescence was first demonstrated using non-luminescent mutants. In particular, mutants containing disruptions in *luxI*, *luxR* or *luxA*, which are as capable as their parental wild-type strain in initiating the symbiosis, have approximately three-fold lower colonization levels (as measured by CFU counts) than wild-type cells at 48 h post-inoculation (p.i.) (Tables 1 and 2) [26,44]. Furthermore, when co-colonized with a wild-type strain at a 1:1 ratio, the *luxA* mutant was outcompeted both at 24 h and 48 h p.i. (Table 3) [44]. Similar colonization results were obtained using a mutant containing an in-frame deletion of *luxCDABEG*, which retains an intact LuxR-LuxI signaling system [45]. The inability of the non-luminous *V. fischeri* strains to persist inside the light organ constitutes a long standing puzzle in the squid-*Vibrio* symbiosis research. However, recent studies are offering useful insights into the puzzle. Luminescence appears to be a “secret language” of communication between the two partners [46]. Luminescence is the second most important signal, after the presence of the symbiont itself, to induce the host gene expression in the light organ [47]. Furthermore, a recent study showed that luminescence is critical for cyclic expression of a cryptochrome gene that encodes a blue-light receptor, which is known to be involved in circadian clock functions in other animals [48]. As the symbiosis initiates and becomes established, *V. fischeri* induces several developmental events in the nascent light organ through bacteria-derived signals called microbe associated molecular patterns (MAMPs), which include the lipid A component of LPS and monomers of peptidoglycan (also referred to as tracheal cytotoxin) [49]. Non-luminous strains show defects in inducing many of these developmental events [44,46,50]. Although these studies illustrate the major role of luminescence in host development, the mechanism by which the squid responds to luminescence is not yet known. Recent evidence of the host’s ability to detect the luminescence has led to the speculation that the squid can recognize and specifically eliminate non-luminous cells from the light organ [51]. The down-regulation of a host gene encoding the blood pigment hemocyanin in the light organ when the host is colonized by the *luxA* mutant [47], suggests that the host may not be able to provide the sufficient oxygen levels for the non-luminous strains that may adversely affect both bacterial growth and light organ development.

While the defects in colonization by the *luxA* or *luxCDABEG* mutants are easily attributed to the disruption of the luciferase enzyme, the corresponding colonization defects of the *luxI* and *luxR* mutants could be due to luminescence-independent functions, as LuxR also regulates 18 non-*lux* genes, including several peptidases and proteases [52,53]. Interestingly, QsrP, which is a putative periplasmic protein encoded by a LuxR-regulated gene, is highly abundant in *V. fischeri* cells associated with adult light organs [54]. Future colonization experiments are required to determine the role of QsrP and other non-*lux* genes in the context of symbiosis.

### 2.2. Impact of AinS-AinR Signaling

Luminescence in *V. fischeri* is thought to be induced sequentially by the multiple QS systems [14]. At intermediate cell density (10^8^–10^9^ cells/mL), the AinS-AinR system induces luminescence 1) by enhancing *luxR* expression via increased levels of LitR and 2) from the direct binding of C8-HSL to LuxR, which forms a complex that can also activate transcription [55]. Within the light organ, where bacterial cell densities can exceed 10^10^ cells/mL, luminescence is predominantly induced by LuxI-LuxR signaling. However, the AinS-AinR system does continue to impact light production in situ, as demonstrated by the reduced luminescence the *ainS* mutant exhibits at 48 h p.i. (Table 1) [14]. Although the reported animal luminescence levels yield large variations, they were routinely less than 40% of those produced by animals colonized by the wild-type strain [14]. Normal animal luminescence levels are restored in a *luxO ainS* double mutant, showing that the effect of the AinS signal on luminescence *in situ* occurs through LuxO [14]. Interestingly, these studies revealed that colonization levels of *luxI*, *ainS*, and *ainS luxI* mutants are comparably reduced at 48 h and 72 h relative to wild type (Table 2) suggesting AinS signaling is as important as LuxI signaling for the persistence of symbionts within the light organ [14]. It is tempting to attribute the colonization defect associated with the *ainS* mutant to the requirement of bacterial luminescence by the host at this stage of symbiosis [44]. However, a luminescence level as low as 10% of that emitted by animals colonized by wild-type cells is sufficient to rescue colonization defects associated with non-luminous strains [44], which suggests AinS also regulates colonization factors that are independent of luminescence. Notably, a *litR* mutant does not impact colonization levels or animal luminescence at 24 or 48 h p.i., which suggests that these AinS-dependent colonization factors are also independent of LitR [16,17]. It may be possible that the colonization factors are encoded by LuxR-regulated, non-*lux* genes, and that AinS impacts expression of those genes through the direct binding of C8-HSL to LuxR. However, further investigations are required to discern the luminescence-independent roles of AinS-AinR signaling in persistence.

AinS-AinR signaling also plays a significant role during the initial steps of host colonization that is distinct from its role in persistence (Tables 1 and 2). At 12 h p.i., the colonization levels of the *ainS* and *litR* mutants are each two-fold lower than wild-type levels [26]. Beyond 24 h, the *litR* mutant displays normal colonization levels and, in fact, out-competes wild-type cells, suggesting that the role of AinS-AinR signaling changes as the symbiosis develops [17,26]. Interestingly, deletion of *luxO* does not restore normal colonization levels in an *ainS* mutant, which has been observed at 72 p.i. [14,26]. It was hypothesized that an appropriate level of LuxO protein is crucial for the proper functioning of signaling [26]. Alternatively, AinS-AinR signaling may only be important at a certain stage of colonization initiation, and constitutive expression of LitR due to the *luxO* mutation has a negative impact.

Microarray experiments designed to compare the transcriptome of the *ainS* mutant with a mutant expressing a constitutively active variant of LuxO (LuxOD47E) revealed multiple genes regulated by the AinS-AinR system that are independent of luminescence [26]. LuxO activates several genes involved in flagella-based motility (e.g., flagellin genes), which is a trait critical for *V. fischeri* to colonize the light organ [39,56,57]. Non-motile mutants are able to form normal aggregate outside of the light organ, but do not migrate into the light organ [58]. Interestingly, hypermotile mutants exhibit colonization defects during both initiation and persistence phases of colonization [39]. More specifically, hypermotile mutants show a delay in both aggregation and migration behaviors important for colonization initiation. Moreover, they colonize the light organ at 10%–50% of wild-type levels at 12 and 24 p.i., (Table 4) demonstrating that the proper regulation of genes involved in motility remains important after the initial colonization event. This general persistence defect associated with hypermotile strains suggests that proper regulation of motility is critical for symbiosis.

The transcriptome analysis described above also revealed that the expression of additional regulatory proteins is controlled by the AinS-AinR system, which highlights the links between the QS network and other regulatory pathways [26]. For instance, the sigma factor RpoQ, which was shown to be repressed by LuxO in the transcriptome analysis, leads to chitinase activity, thereby linking QS to the ability of *V. fischeri* to break down chitin [59]. Intriguingly, RpoQ also represses both luminescence and motility suggesting that high levels of RpoQ can counteract the ability of LitR to activate *luxR* expression.

### 2.3. Impact of the LuxS/LuxP/Q Signaling

In contrast to a mutation in *ainS*, mutation of *luxS* alone does not affect colonization levels at 12, 24, or 48 h p.i. (Table 2) [25,26]. In addition, the level of the *ainS luxS* double mutant at 12 h p.i. is comparable to the *ainS* mutant [26]. A synergistic effect from the two signaling pathways only manifests at 24 h p.i., as demonstrated by the reduced colonization level of the double mutant compared to the *ainS* mutant [25]. Interestingly, the *luxS* mutant colonizes the host normally when inoculated with wild-type cells [25]. It was hypothesized that the signal secreted by wild-type cells rescues any colonization defect associated with the *luxS* mutant. In contrast, the defects associated with mutations in either *ainS* and *luxI* continue to manifest in competition experiments involving the wild-type strain [14,44].

## 3. Influence of Abiotic Environment Cues on the QS Network

Wild-type *V. fischeri* ES114 produces a level of luminescence in culture that is three orders of magnitude lower than that achieved within the light organ [60]. This observation has been attributed in part to differences in the cell density between the light organ and culture environments that impact the level of 3-oxo-C6-HSL. The addition of exogenous 3-oxo-C6-HSL induces culture luminescence to a level comparable to that observed in the light organ [44]. The low level of the 3-oxo-C6-HSL in culture is puzzling considering a positive feedback loop amplifies synthesis of the autoinducer. However, an alternative explanation is that environmental factors other than population density impact the synthesis of autoinducers [61]. For instance, recent studies have suggested conditions of low iron in the host may affect luminescence by regulating components of the QS network [62,63]. In particular, low iron conditions lead to increased luminescence by removing Fur-mediated repression of *litR* expression [63]. Luminescence of wild-type *V. fischeri* is enhanced under reduced levels of inorganic phosphate (P_i_), and this effect is lost in a *phoB* mutant [64]. The *phoB* gene encodes a response regulator that is phosphorylated by the sensor PhoR under low P_i_ conditions. These results suggest that the effect of P_i_ levels is mediated by the PhoR/PhoB two-component system [65]. Luminescence is also repressed by oxygen as a result of the two-component system ArcA-ArcB [45,66]. In fact, de-repression by the ArcA-ArcB system leads to an increase in luminescence that depends on the presence of the positive feedback loop associated with *luxI* [66].

## 4. Concluding Remarks and Future Perspective

**Figure 3:**
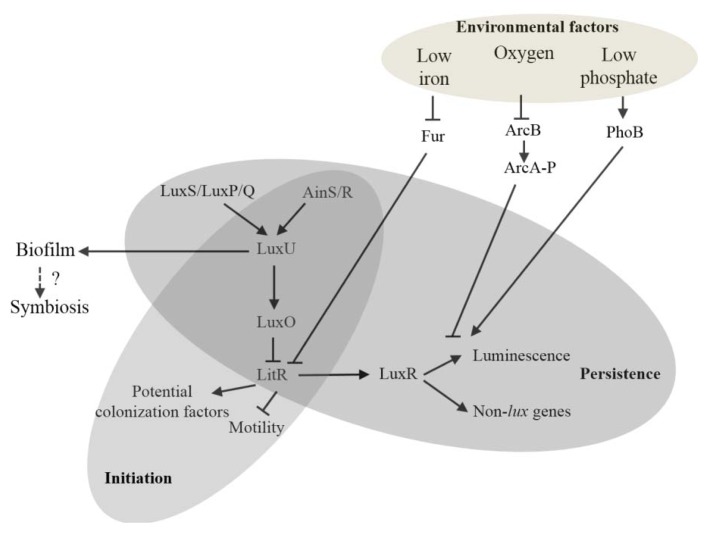
Role of the QS network of *V. fischeri* in symbiosis with *E. scolopes*. The QS network of *V. fischeri*, which is formed by three interconnected QS systems, regulates the factors that *V. fischeri* uses to initiate and persist in a mutualistic symbiosis with *E. scolopes*. Motility and other unknown factors are regulated by the AinS-AinR system and play important roles in the initial steps of host colonization. Luminescence, which is controlled directly by the LuxI-LuxR system and indirectly by the AinS-AinR and LuxS-LuxP/Q systems, is required for bacterial persistence. Various environmental signals integrate into the QS network affecting the luminescence output. The QS network is also linked to other regulatory pathways controlling bacterial activities such as biofilm formation, whose functions are unclear in the symbiosis.

Together, the studies described above highlight the complexity of QS in *V. fischeri*. The LuxI-LuxR, AinS-AinR, and LuxS-LuxP/Q QS systems form an interconnected signaling network that *V. fischeri* uses to regulate various factors involved in the squid-*Vibrio* symbiosis (Figure 3). While regulation of light production by QS has received substantial attention, the control by this regulatory network over many other bacterial activities, including motility, chitinase activity, biofilm formation, and acetate metabolism, is also an interesting direction of research. All three QS systems affect bacterial persistence through their ability to regulate luminescence production. In contrast, only AinS-AinR signaling impacts the initial steps of colonization, primarily through the regulation of motility and other uncharacterized factors by LitR. The QS network has also evolved to respond to various environmental conditions, such as the levels of oxygen, iron, and phosphate. The *Euprymna scolopes-Vibrio fischeri* symbiosis will continue to elucidate the various functions of quorum sensing in the context of host colonization.

## Figures and Tables

**Table 1:** Bioluminescence levels emitted by animals colonized by various *V. fischeri* mutants. The luminescence levels of squid colonized by mutant strains are relative to those of animals colonized by a wild-type *V. fischeri* strain, which is defined as 100%. The luminescence levels shown in the table for some of the animals are approximate, and we therefore refer readers to the original studies for more details.

Strain	Luminescence at different stages of symbiosis				References

	12 h	24 h	48 h	72 h	
*ainS*	10%–20%	10%–20%	10%–40%	ND	[14,25]
*luxO*	100%	100%	100%	ND	[14]
*ainS*, *luxO*	100%	100%	100%	ND	[14]
*luxS*	100%	100%	ND	ND	[25]
*luxS*, *ainS*†	50%	50%	ND	ND	[25]
*litR*	100%	100%	100%	ND	[17]
*luxR*	BD	BD	ND	ND	[44]
*luxI*	BD	BD	ND	ND	[44]
*luxA*	BD	BD	ND	ND	[44]

† Luminescence is relative to the *ainS* mutant instead of the wild type strain;

ND = Not Determined; BD = Below Detection.

**Table 2:** Bacterial levels in animals colonized by various *V. fischeri* mutants. The bacterial loads of squid colonized by mutant strains are relative to those of animals colonized by a wild-type *V. fischeri* strain, which is defined as 100%. The colonization levels shown in the table for some of the animals are approximate, and we therefore refer readers to the original studies for more details.

Strain	Colonization level at different stages of symbiosis				References

	12 h	24 h	48 h	72 h	
*ainS*	45%	75%	40%	20%	[14,26]
*luxO*	37%	ND	ND	100%	[14,26]
*LuxO(D47E)*	52%	ND	ND	97%	[26]
*ainS*, *luxO*	36%	ND	ND	100%	[14,26]
*luxS*	95%	75%	90%	ND	[25,26]
*luxS*, *ainS*	48%	50%	20%	ND	[25,26]
*litR*	51%	100%	100%	ND	[17,26]
*luxR*	119%	100%	25%–35%	ND	[26,44]
*luxI*	115%	100%	25%–35%	ND	[26,44]
*luxA*	ND	100%	25%–35%	ND	[44]
*luxCDABEG*	ND	ND	25%–35%	ND	[45]
*ainS*, *luxI*	79%	75%	30%	20%	[14,26]

ND = Not Determined.

**Table 3:** Competition advantage of different *V. fischeri* mutants in co-colonization experiments.

Strains in mixed inoculums (1:1 ratio)		Dominant strain at different stages of symbiosis			References
	
Strain 1	Strain 2	12 h	24 h	48 h	
ESR1	*luxA*	ND	ESR1	ESR1	[44]
ESR1	*luxR*	ND	-	ESR1	[44]
ES114	*litR*	ND	ND	*litR*	[16,17]
ES114	*qrr1*	ND	ND	ES114	[16]
ES114	*luxO*	ND	ND	ES114	[16]
ES114	*luxO*, *litR*	ND	ND	None	[16]
ES114	*qrr1*, *litR*	ND	ND	None	[16]
ES114	*ainS*	ND	ND	ES114	[14]
ES114	*luxCDABEG*	ND	ND	ES114	[45]

ND = Not Determined.

**Table 4:** Motility-associated phenotypes, aggregation behaviors, and colonization levels of various *V. fischeri* mutants.

Strain	Motility behavior on agar		Flagellation	Aggregation behavior	Colonization level at different stages of symbiosis			Reference
	
	0.3%–0.7%	0.25%			12 h	24 h	48 h	
ES114	Motile	Motile	Normal flagella	Normal	100%	100%	100%	[56]
N210	Non-motile	ND	Normal flagella	ND	ND	BD	ND	[56]
NF201	Non-motile	ND	No flagella	ND	ND	BD	ND	[56]
NM200	Non-motile	ND	Abnormal flagella	ND	ND	BD	ND	[56]
DM66	Hypermotile	ND	Hyper flagellation	Delayed	50%	60%	100%	[39]
DM73	Hypermotile	ND	Hyper flagellation	Delayed	ND	40%	ND	[39]
DM61	Hypermotile	ND	Hyper flagellation	Delayed	0.1%–10%	0.1%–10%	0.1%–10%	[39]
*flaA*	ND	Less motile	Hypo flagellation	Normal	ND	20%–25%	ND	[58]
*flrA*	Non-motile	ND	No flagella	Normal	ND	BD	ND	[58]
*ainS*	Motile	Hyper-motile	ND	Normal				[26]
*litR*	ND	Hyper-motile	ND	ND				[26]
*luxO*	ND	Non-motile	ND	ND		See Table 1		[26]
*luxR*	ND	Motile	ND	ND				[26]
*luxI*	Motile	Motile	ND	ND				[26]

ND = Not Determined; BD = Below Detection.
